# Curcuphenol possesses an unusual histone deacetylase enhancing activity that counters immune escape in metastatic tumours

**DOI:** 10.3389/fphar.2023.1119620

**Published:** 2023-08-10

**Authors:** Samantha L. S. Ellis, Sarah Dada, Lilian L. Nohara, Iryna Saranchova, Lonna Munro, Cheryl G. Pfeifer, Brett A. Eyford, Tunc Morova, David E. Williams, Ping Cheng, Nathan A. Lack, Raymond J. Andersen, Wilfred A. Jefferies

**Affiliations:** ^1^ Michael Smith Laboratories, University of British Columbia, Vancouver, BC, Canada; ^2^ Centre for Blood Research, University of British Columbia, Vancouver, BC, Canada; ^3^ The Djavad Mowafaghian Centre for Brain Health, University of British Columbia, Vancouver, BC, Canada; ^4^ Department of Microbiology and Immunology, University of British Columbia, Vancouver, BC, Canada; ^5^ Vancouver Prostate Centre, Vancouver Coastal Health Research Institute, Vancouver, BC, Canada; ^6^ Departments of Medical Genetics, Zoology, and Urologic Sciences, University of British Columbia, Vancouver, BC, Canada; ^7^ Departments of Chemistry and Earth Ocean, and Atmospheric Sciences, University of British Columbia, Vancouver, BC, Canada; ^8^ School of Medicine, Koç University, Istanbul, Türkiye

**Keywords:** epigenetic modification, antigen processing machinery, curcuphenol, major histocompatibility complex class I, histone deacetylase activity, HDAC, tumours, drug discovery

## Abstract

Curcuphenol, a common component of the culinary spices, naturally found in marine invertebrates and plants, has been identified as a novel candidate for reversing immune escape by restoring expression of the antigen presentation machinery (APM) in invasive cancers, thereby resurrecting the immune recognition of metastatic tumours. Two synthetic curcuphenol analogues, were prepared by informed design that demonstrated consistent induction of APM expression in metastatic prostate and lung carcinoma cells. Both analogues were subsequently found to possess a previously undescribed histone deacetylase (HDAC)-enhancing activity. Remarkably, the H3K27ac ChIPseq analysis of curcuphenol-treated cells reveals that the induced epigenomic marks closely resemble the changes in genome-wide pattern observed with interferon-γ, a cytokine instrumental for orchestrating innate and adaptive immunity. These observations link dietary components to modifying epigenetic programs that modulate gene expression guiding poised immunity.

## Introduction

Cancer is a devastating disease that arises from genetic mutations and epigenetic modifications that govern cell proliferation, apoptosis, senescence, and invasion (Alimonti et al., 2000; Setiadi et al., 2005a; Nguyen and Massagué, 2007a; Setiadi et al., 2007a; Esteller, 2008; Setiadi et al., 2008a; Seliger et al., 2012; Saranchova et al., 2016; Saranchova et al., 2018; Dhatchinamoorthy et al., 2021a; Dada et al., 2023; Nohara et al., 2023). A common signature shared by invasive forms of metastatic cancers originating from many different tissues, is the loss of immunogenicity that consequently results in immune evasion (Alimonti et al., 2000; Shankaran et al., 2001; Setiadi et al., 2005a; Lou et al., 2007; Setiadi et al., 2007a; Setiadi et al., 2008a; Saranchova et al., 2016; Saranchova et al., 2018; Dhatchinamoorthy et al., 2021a; Dada et al., 2023; Nohara et al., 2023). This can be achieved through several mechanisms, one of which involves evasion through the loss of the antigen presentation machinery (APM), a process known as immune escape or immunoediting. In this neo-Darwinian process, the strong negative selective pressure by adaptive immune surveillance mechanisms against emerging primary cancers can result in the selection of tumour variants that have lost the expression of APM genes, rendering subsequent metastasis invisible to recognition by host T lymphocytes (Jefferies et al., 1993a; Gabathuler et al., 1994; Alimonti et al., 2000; Shankaran et al., 2001; Setiadi et al., 2007a; Saranchova et al., 2018; Tang et al., 2020; Dhatchinamoorthy et al., 2021a). Under normal physiological conditions, APM presents bound self and foreign peptides via the major histocompatibility class I (MHC-I) molecules to cytotoxic T lymphocytes (CTLs) of the adaptive immune system (Jefferies et al., 1993a; Alimonti et al., 2000; Setiadi et al., 2005a; Saranchova et al., 2016). To generate these peptides, endogenously expressed proteins are degraded via proteasomes in the cytosol before being conveyed into the endoplasmic reticulum (ER) by the transporters associated with antigen processing 1 and 2 (TAP-1/2). In the ER, the peptides are loaded onto the MHC-I molecules before being transported to the cell surface (Gabathuler et al., 1994; Brucet et al., 2004; Lou et al., 2007; Setiadi et al., 2008a). Upon interaction of the CTLs with the MHC-I peptide complexes, the CTLs are able to distinguish between normal, cancerous, or pathogen-infected cells (Jefferies et al., 1993a; Gabathuler et al., 1994; Alimonti et al., 2000; Setiadi et al., 2005a; Saranchova et al., 2016), resulting in the initiation of the appropriate immune response and the subsequent destruction of the cancerous or pathogen-infected cells (Jefferies et al., 1993b; Gabathuler et al., 1994; Lankat-Buttgereit and Tampe, 2002). However, this normal mechanism is often subverted in invasive metastatic tumours.

While the loss of the endogenous APM components is widely seen in metastatic cancers, the exact mechanisms leading to the reduction of APM components have yet to be fully explored. Previous work in the field has shown that MHC-I expression can be re-established in metastatic cancer through the use of cytokines such as interferon-γ (IFN-γ) (Jefferies et al., 1993a; Gabathuler et al., 1994; Alimonti et al., 2000; Diedrich et al., 2001; Shankaran et al., 2001) and interleukin-33 (Saranchova et al., 2016) or by genetic complementation with the missing APM components (Gabathuler et al., 1994; Alimonti et al., 2000; Setiadi et al., 2005a; Saranchova et al., 2016). Stimulation with IFN-γ, has shown great success in inducing the expression of the APM *in vitro* (Jefferies et al., 1993a; Gabathuler et al., 1994; Johnsen et al., 1998; Setiadi et al., 2005a; Setiadi et al., 2007a), however, its therapeutic translation into therapeutics remains problematic, and therefore, therapies targeting up-stream regulators need to be explored.

Histone acetylation is a major epigenetic modification that affects gene transcription and is mediated by histone acetyltransferases (HATs) and histone deacetylases (HDACs) (Ruijter et al., 2003; Gregoretti et al., 2004). HATs acetylate lysine residues on histone proteins, resulting in relaxation of chromatin structure that facilitates gene induction. Conversely, HDACs remove acetyl groups from hyperacetylated histones and reduces general gene transcription. Non-histone proteins can also be acetylated and deacetylated. Based on phylogenetic sequence analysis and homology to yeast HDACs, HDACs are classified into class I (HDACs 1–3 and −8), class II (HDACs 4–7 and −9), class III (HDAC), also known as sirtuins (SIRT1–7), and class IV (HDAC11) (Ruijter et al., 2003; Gregoretti et al., 2004). HDACs play crucial roles in biological processes including cell proliferation, apoptosis, inflammation, and in molecular processes associated with cancer (Chen et al., 2015; Li et al., 2021; Song et al., 2021; Xu et al., 2021; Borcoman et al., 2022). Additionally, we previously discovered that treatment with HDAC inhibitors resulted in the re-establishment of the APM expression and function and also reduces tumour growth *in vivo*, surprisingly indicating that the loss of the APM expression can be reversed (Setiadi et al., 2005a; Setiadi et al., 2007a; Setiadi et al., 2008a; Saranchova et al., 2016; Saranchova et al., 2018; Dada et al., 2023; Nohara et al., 2023).

Previously, we demonstrated that functional deficiencies of APM can be restored through genetic complementation by introducing the TAP-1 gene or TAP-1 and TAP-2 genes under the control of heterologous promoters into metastatic tumours by transfection or by recombinant virus infection of tumour-bearing mice that resurrects CTL recognition and reduces invasive tumour growth and metastasis *in vivo* (Gabathuler et al., 1994; Alimonti et al., 2000). Intriguingly, we also found that the TAP-1 deficiency was not regulated by mutations or other defects in the TAP-1 gene in many metastatic tumours, but it was epigenetically regulated (Setiadi et al., 2007b) and could be restored by treatment with HDAC inhibitors, such as trichostatin-A (TSA) (Setiadi et al., 2008a). Furthermore, our original study have demonstrated that although TSA promotes differentiation, cell cycle arrest and apoptosis in tumour cells (Peixoto and Lansiaux, 2006), it is not effective in decreasing tumour growth in TSA-treated nude mice (Setiadi et al., 2008a), which lack functional T-lymphocytes. These findings strongly suggest that in TSA-treated animals, the immune recognition of tumours is increased and that the TSA effect is mediated by increasing the adaptive immune response *in vivo* (Setiadi et al., 2008a). Although TSA has been shown to confer anti-cancer effects *in vitro* and *in vivo* (Marks et al., 2001; Zhu and Otterson, 2003; Mukhopadhyay et al., 2006), cancer treatments using HDAC inhibitors can be inefficient due to their instability and low retention *in vivo* (Monneret, 2005). This limitation may be overcome by the development of non-toxic compounds that can lead to the modification of the epigenome, that are more stable *in vivo*, and improve efficacy in inducing immune recognition of invasive tumours.

In this context, we sought to study the molecular mechanisms of action of curcuminoid compounds such as curcuphenol. Curcuphenol naturally occurs in plants and some marine sponges and is a component of culinary spices including cumin and turmeric that are used in medicinal ethnobiology and ethnopharmacology as traditional medicines and as dietary supplements (Paul and Sa, 2021; Dada et al., 2023). We previously discovered that cannabinoids and curcuphenol are able to reverse the immune escape phenotype of metastatic tumours *in vitro* (Dada et al., 2023; Nohara et al., 2023). Here we test the hypothesis that curcuphenol and synthetic analogues of curcuphenol, possess a previously undescribed HDAC inhibitory activity, that reverses immune escape by resurrecting APM expression in metastatic tumours and therefore, underpins curcuphenol’s anti-metastasis activity. The results of these experiments, however, disprove this hypothesis and support a more intriguing mechanism of action for curcuphenol.

## Results

### MHC-I and TAP-1 expression in TC1 primary cells and A9 metastatic cells

**FIGURE 1:**
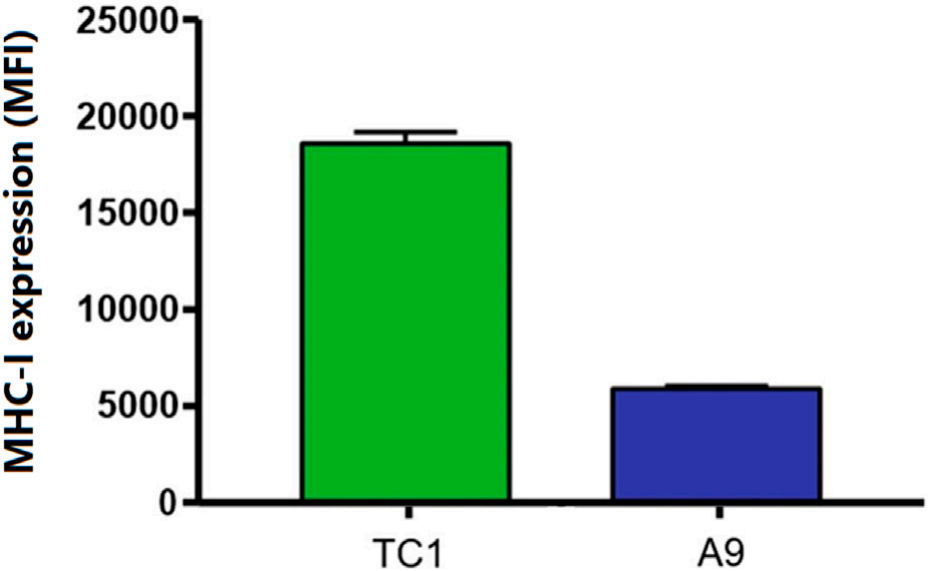
A common immune-escape phenotype is illustrated where MHC-I expression is higher in primary tumours compared to metastatic tumours. Cell surface MHC-I expression in TC1 primary cells and A9 metastatic cells as demonstrated by flow cytometry analysis. Mean fluorescence intensity (MFI).

**FIGURE 2:**
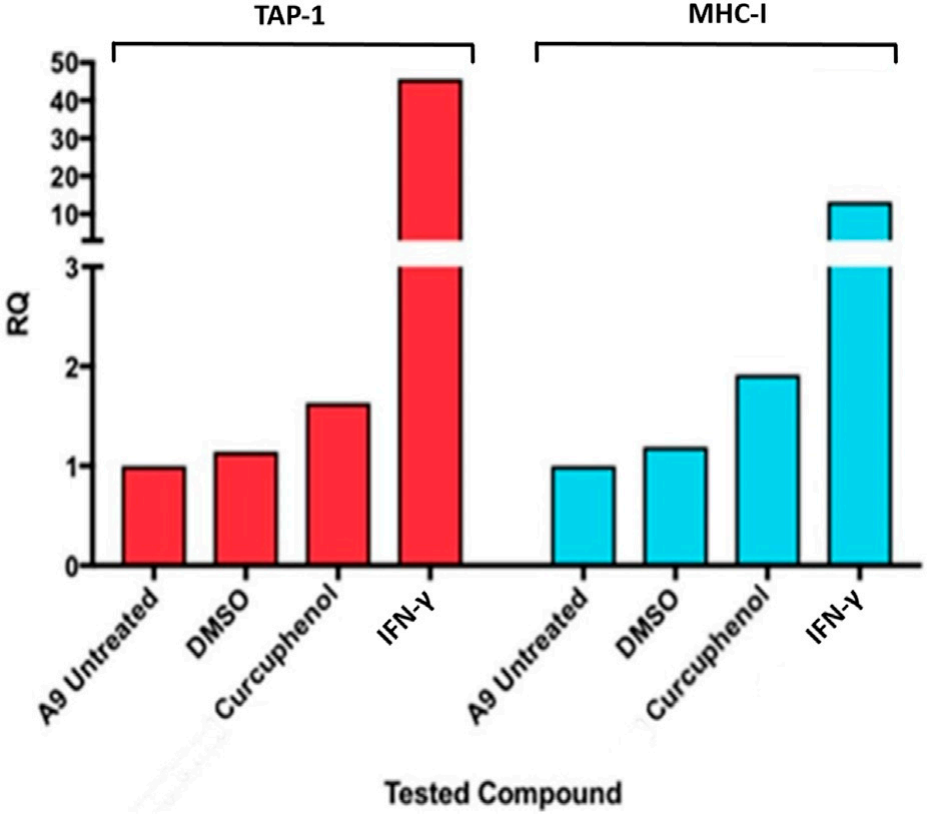
Curcuphenol induces MHC-I and TAP-1 mRNA expression in A9 metastatic tumour cells. Treatment of A9 metastatic cells with curcuphenol (0.02 mg/mL) and IFN-γ (5.832 × 10^−6^ nmol) for 48 h induce increased MHC-I and TAP-1 mRNA expression as assessed by RT-PCR.

One method by which metastatic cancer evades the immune system is through genetic or epigenetic mechanisms resulting in the loss of expression of endogenous APM components (Alimonti et al., 2000; Shankaran et al., 2001; Setiadi et al., 2005a; Setiadi et al., 2007a; Setiadi et al., 2008a; Saranchova et al., 2016; Saranchova et al., 2018; Dada et al., 2023). The observation that MHC-I expression is reduced in A9 metastatic cell lines compared to TC1 primary cell lines (Schnare et al., 2001; Jones and Baylin, 2002; Reiman et al., 2007) (Figure 1) points to loss of expression of MHC-I and other APM components as a mechanism of immune evasion by metastatic cancer. We previously used a high-throughput cell-based screening assay to identify chemical entities that reverse the downregulation of APM components in cell lines derived from metastatic tumours. We discovered that one of these entities, curcuphenol, induces the expression of the APM components, TAP-1 and MHC-I molecules, in cell lines derived from both metastatic prostate and lung carcinomas (Nohara et al., 2023). We therefore treated A9 metastatic cells with curcuphenol and IFN-γ and analyzed them via RT-PCR. We found that both curcuphenol and IFN-γ induce the expression of MHC-I mRNA and the APM component TAP-1 mRNA in A9 metastatic cells (Figure 2), thereby reversing an immune escape phenotype in these cells.

### Isolation, identification, and synthesis of curcuphenol and its synthetic analogues

**FIGURE 3:**
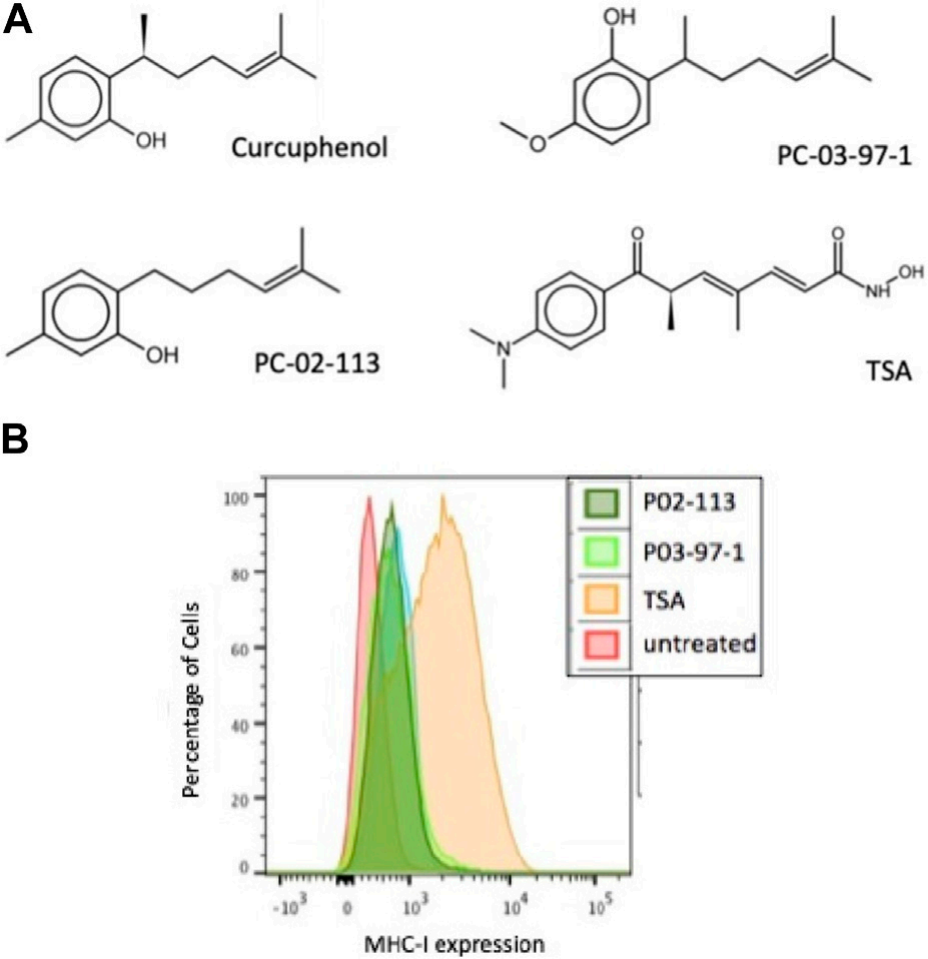
The curcuphenol analogues, P02-113 and P03-97-1, induce MHC-I expression in A9 metastatic tumour cells. **(A)** Structures of curcuphenol, TSA and two synthesized analogues, P02-113 and P03-97-1 **(B)** A9 metastatic cells were treated for 48 h with P02-113 (0.02 mg/mL), P03-97-1 (0.02 mg/mL) or TSA (100 ng/mL). Cell surface MHC-I expression was subsequently assessed by flow cytometry. Three experimental replicates and three technical replicates were performed for each experiment. The figure shows data from one of the experimental replicates.

Based upon these results, we synthesized curcuphenol analogues to test their potential as immunotherapeutic anti-cancer agents. A series of lower CLogP and achiral analogues that had structural modifications on the phenol ring and the carbon tail were synthesized and assessed for their ability to induce MHC-I expression *in vitro*. From this small synthetic library, two analogues, P02-113 and P03-97-1 (Figure 3A), were tested and showed consistent induction of MHC-I expression at the cell surface 48 h after treatment when measured by flow cytometry (Figure 3B).

### Effects of P02-113 and P03-97-1 on class I/II histone deacetylase activity

**FIGURE 4:**
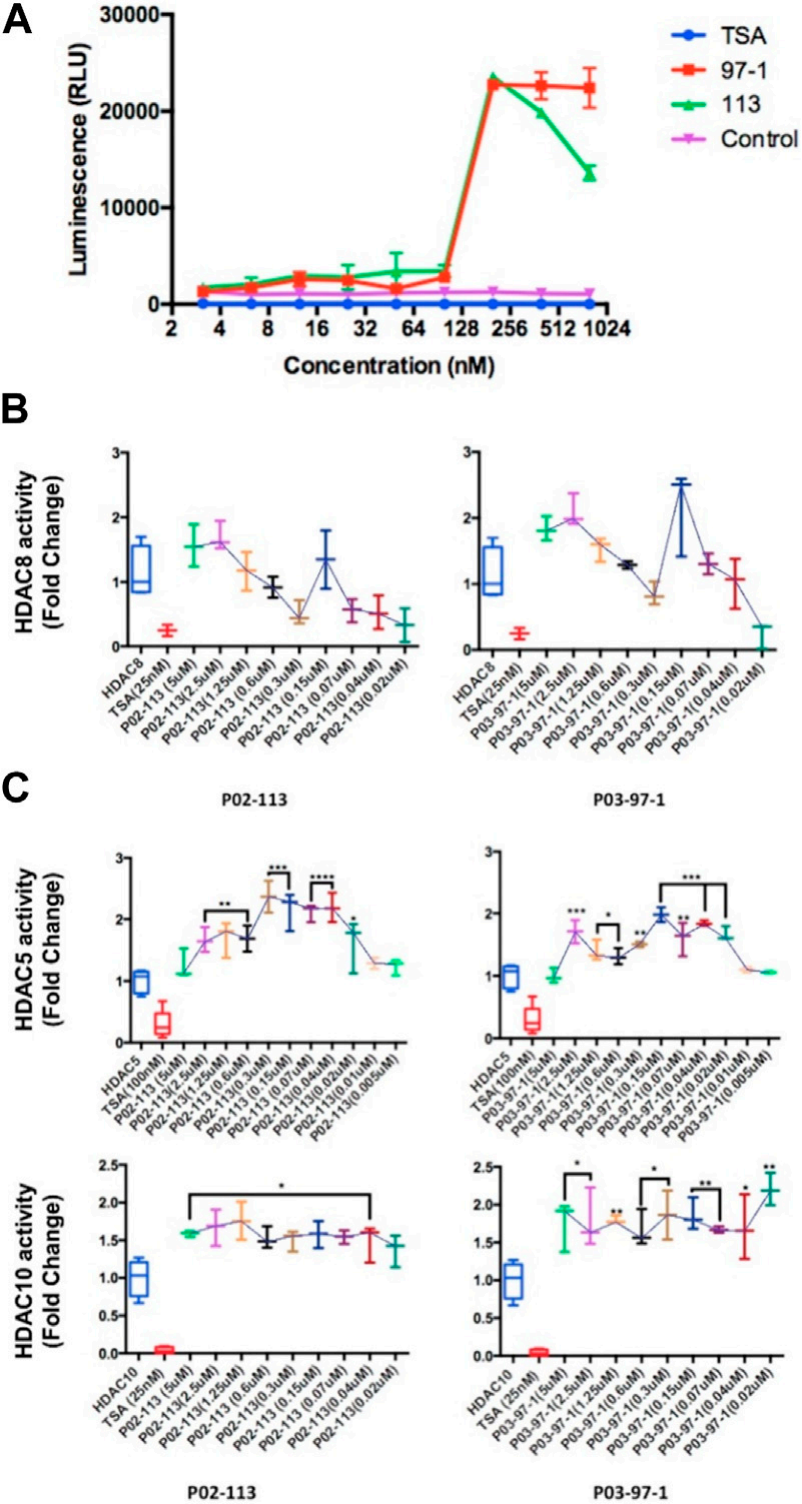
Effects of P02-113 and P03-97-1 on HDAC class I/II histone deacetylase activity. **(A)** Class I/II HDAC activity was assessed using the HDAC-GloTM I/II Assay and Screening System (Promega) in metastatic A9 cells after treatment with P02-113 or P03-97-1. A9 cells were treated with vehicle, TSA (50 nM), or a range of concentrations of P02-113 or P03-97-1. Fluorescence was measured using the Infinite M200 (Tecan) with i-control software (Tecan). **(B)** HDAC8, a class I HDAC, showed a change in activity when exposed to P02-113 or P03-97-1. HDAC8 was the only HDAC that showed slight inhibition at lower concentrations for both compounds. **(C)** Class II HDAC assay of HDACs with enhanced activity upon treatment with either P02-113 or P03-97-1. HDACs 5 and 10 were the only class II HDACs showing an increase in activity levels upon treatment with curcuphenol analogues. **** = most significant change in activity; * = lowest significant change in activity using a Student’s t-test. Three experimental replicates and three technical replicates were performed for each experiment. The figures show data from one of the experimental replicates.

Due to the structural similarity of the curcuphenol analogues, P02-113 and P03-97-1, to a previously described HDAC inhibitor, TSA, it was hypothesized that these molecules could be acting through a similar mechanism. To test this hypothesis, we evaluated the ability of a cell-based HDAC assay that uses a cell-permeable HDAC I/II substrate that can be converted into a luciferase substrate upon deacetylation by endogenous HDACs, and therefore no wash steps are needed in the assay. We evaluated the ability of P02-113 and P03-97-1 to affect the activity of class I/II HDACs. Interestingly, the compounds P02-113 and P03-97-1 exhibited the opposite effect to what was hypothesized and showed an increase in class I/II HDAC activity (Figure 4A). Even at the lowest concentrations of 1–100 nM, HDAC activity was induced not inhibited. Both compounds showed a peak in HDAC activity around 180 nM, while P02-113 started to reduce the effect at higher concentrations. P03-97-1 maintained peak levels of HDAC activity until the highest concentration of 1 μM suggesting a stronger effect. The stronger effect exhibited by P03-97-1 could be due to several factors including stronger binding affinities to HDAC enzymes, or better ability to enter A9 cells, however the exact reason remains to be determined.

**FIGURE 5:**
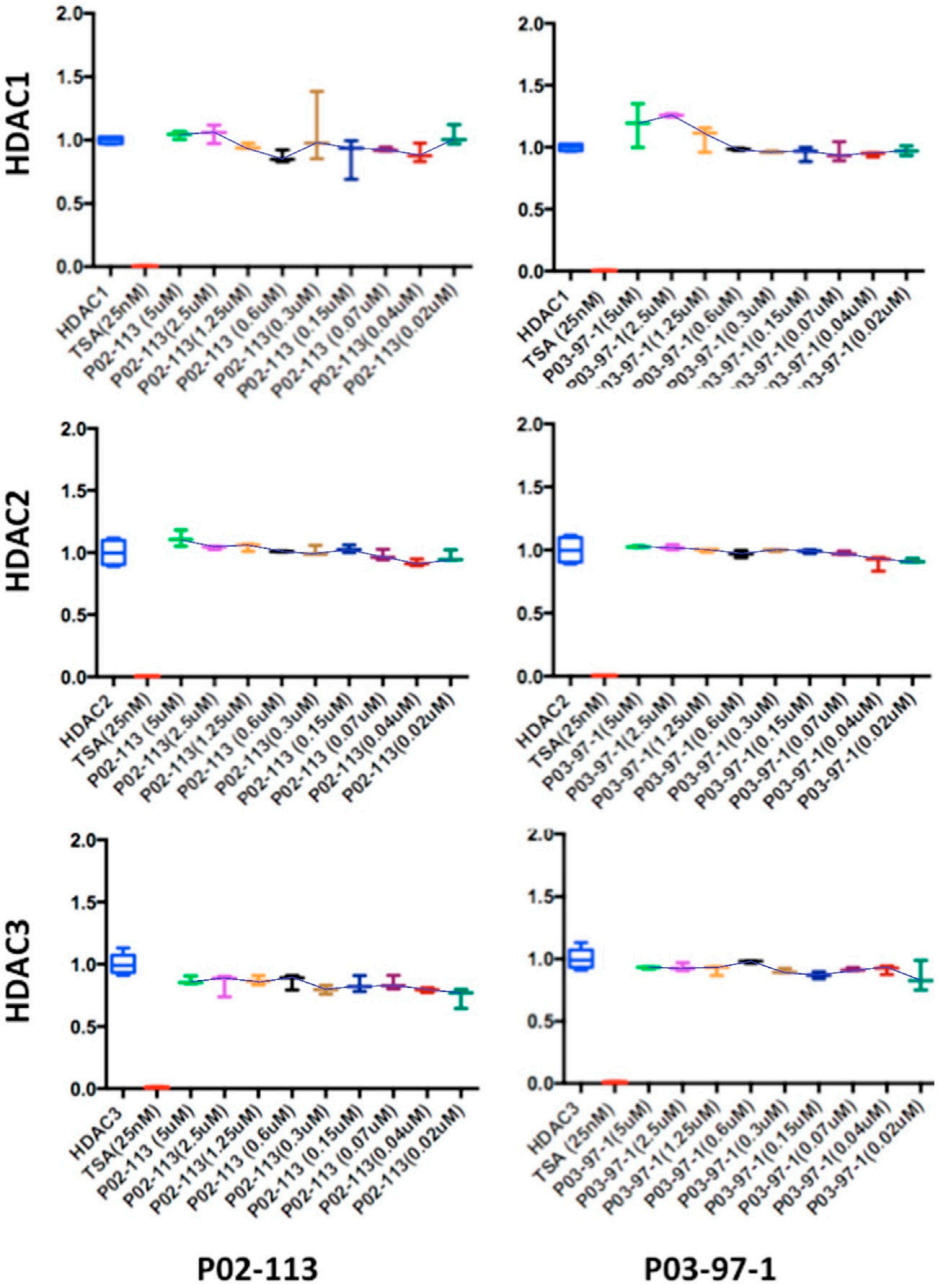
Class I HDAC enzymes are unaffected by P02-113 or P03-97-1. The activities of class I HDACs were evaluated after treatment with P02- 113 or P03-97-1 using the respective HDAC fluorogenic kits (BPS Biosciences). HDACs 1-3 showed no significant change in activity upon treatment with either P02-113 or P03- 97-1 at concentrations ranging from 0.02 to 5 μM using a Student’s t-test. Fold change from control (TSA) is shown on the *Y*-axis. Three experimental replicates and three technical replicates were performed for each experiment. The figures show data from one of the experimental replicates.

Next, the activity of the curcuphenol analogues on individual purified recombinant HDACs was evaluated. Here a fluorogenic HDAC assays designed to measure histone deacetylase activity was used. In addition, we used a potent HDAC inhibitor, TSA, as a positive control. This assay uses a HDAC fluorometric substrate, containing an acetylated lysine side chain, which was then incubated with purified HDAC. The deacetylation sensitizes the substrate so subsequent treatment with the lysine developer solution produces a fluorophore that can then be measured using a fluorescence reader. No significant change in the activities of the class I HDACs 1, 2, and 3 were observed at the concentrations tested for both P02-113 and P03-97-1 (Figure 5). For compound P02-113, the class I HDAC8 showed more variable results with no change in HDAC8 activity at higher concentrations, however, at a concentration of 0.3 uM and below, inhibition was observed, that was similar to the HDAC inhibition exhibited by TSA (Figure 4B). P03-97-1 followed a similar pattern with no change in activity at higher concentrations but at the lowest concentration 0.02uM an inhibitory phenotype was seen. This indicates that the analogues P02- 113 and P03-97-1 act as inhibitors to HDAC8 but not for other HDAC class I enzymes. Another interesting factor that correlates with the inhibitory effects of P02-113 and P0-3-97-1, is that HDACs 1-3 are limited to the nucleus whereas HDAC8 is the only HDAC class I also found in the cytosol.

**FIGURE 6:**
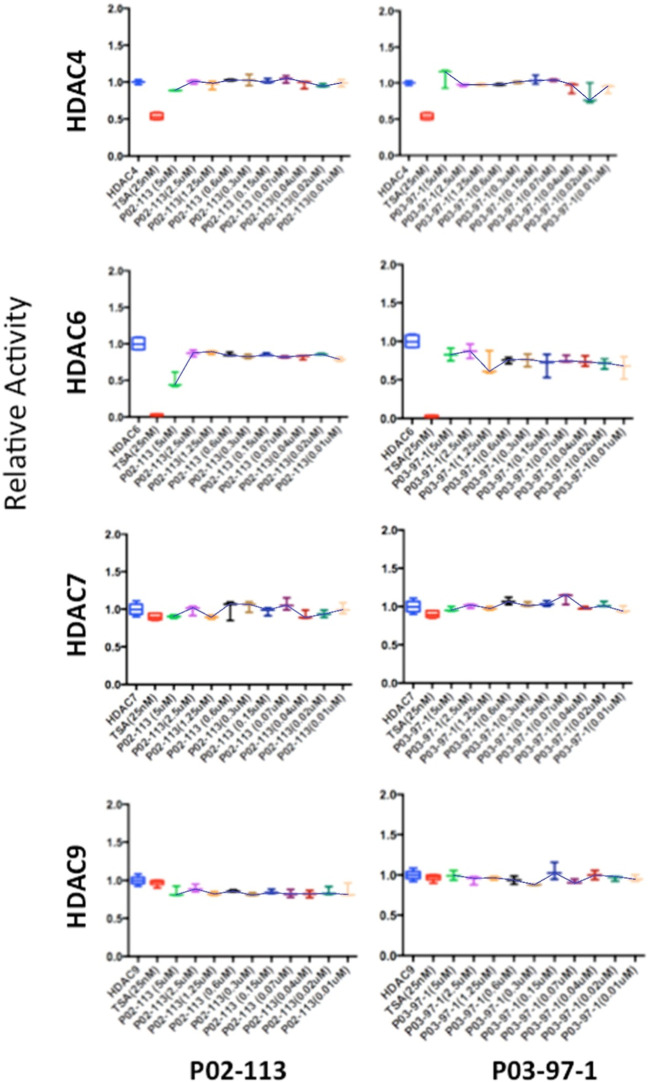
Class II HDAC enzymes are unaffected by P02-113 or P03-97-1. HDACs 4,6,7 and 9 did not show a significant difference in mean activity upon treatment with analogues between the concentrations 0.02–5 μM, as determined by a Student’s t-test. HDACs 4, 6, 7, and 9 were measured using the respective HDAC fluorogenic kits (BPS Biosciences). Fold change from control (TSA) is shown on the *Y*-axis.

**FIGURE 7:**
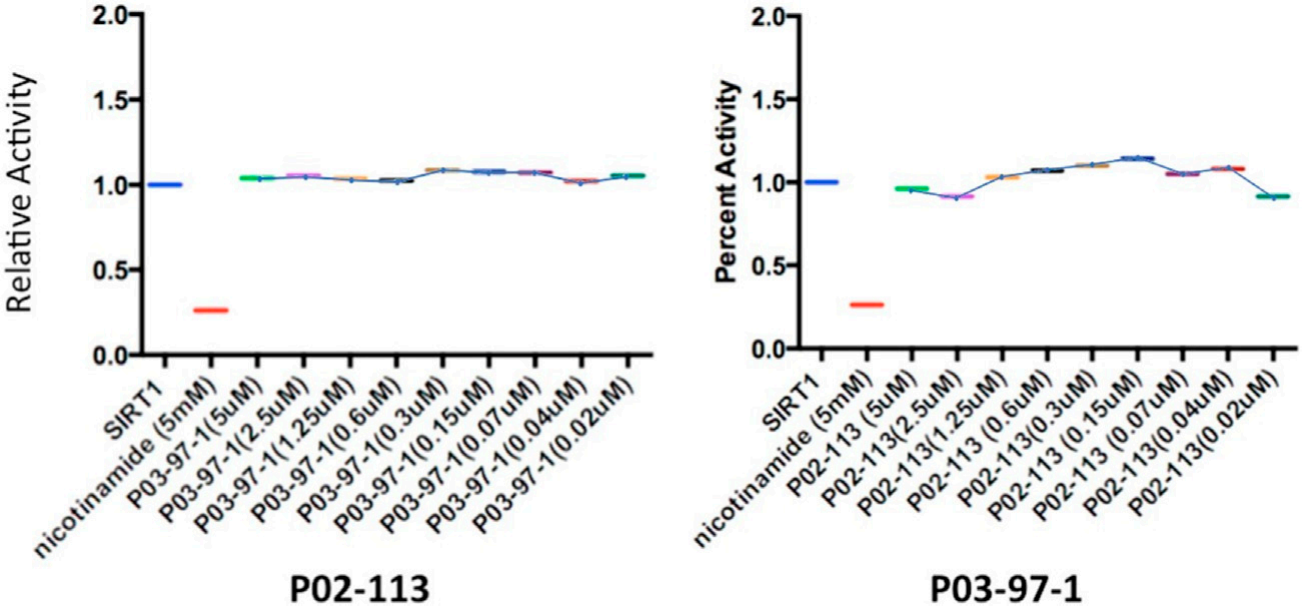
Analysis of SIRT1 activity, from the class III HDAC family, after treatment with P02-113 or P03-97-1. SIRT1 activity was measured using the SIRT1 fluorogenic kit (BPS Biosciences). SIRT1 showed no change in activity upon treatment with P02-113 or P03-7-1 between the concentrations of 0.02–5 μM as determined by a Student’s t-test. Three experimental replicates and three technical replicates were performed for each experiment. The figures show data from one of the experimental replicates. Fold change from control (TSA) is shown on the Y-axis.

**FIGURE 8:**
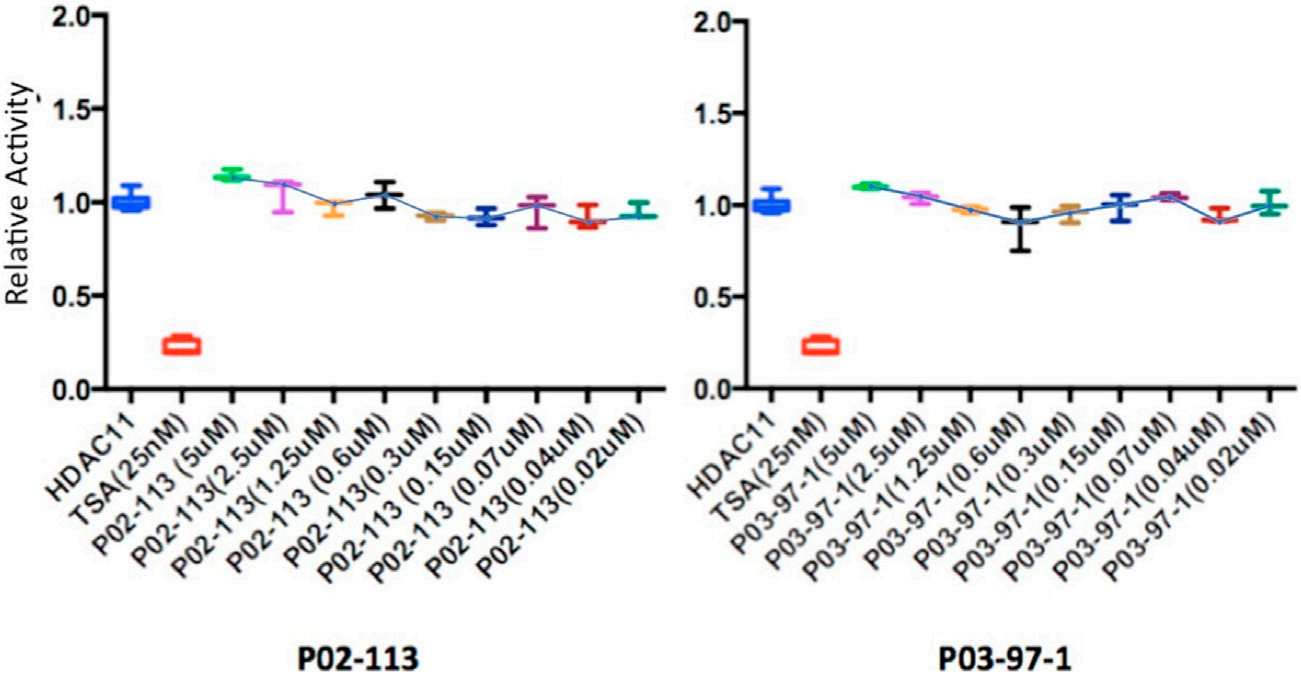
Class IV HDAC activity (HDAC11) was unaffected after treatment with P02-113 or P03-97-1. Mean activity with standard error of HDAC11 was measured upon treatment with P02-113 or P03-97-1 between the concentrations 0.02–5 μM with no significant change seen using a Student’s t-test. HDAC11 activity was measured using the HDAC-GloTM I/II Assay and Screening System (Promega). Three experimental replicates and three technical replicates were performed for each experiment. The figures show data from one of the experimental replicates. Fold change from control (TSA) is shown on the Y-axis.

The HDAC class II family encompasses HDACs 4 through 10 excluding HDAC8. P02-113 and P0-3-97-1 did not affect the activity of the class II HDACs 4, 6, 7, and 9 (Figure 6). Replicate experiments were conducted on all the extracts to achieve these final results. On the other hand, the activities of both HDACs 5 and 10 were significantly enhanced upon treatment with the curcuphenol analogues (Figure 4C). Additionally, no effect was observed in the activities of the HDAC class III enzyme SIRT1 (Figure 7) nor on the HDAC class IV enzyme HDAC11 (Figure 8).

### Functional characterization of HDAC activity

**FIGURE 9:**
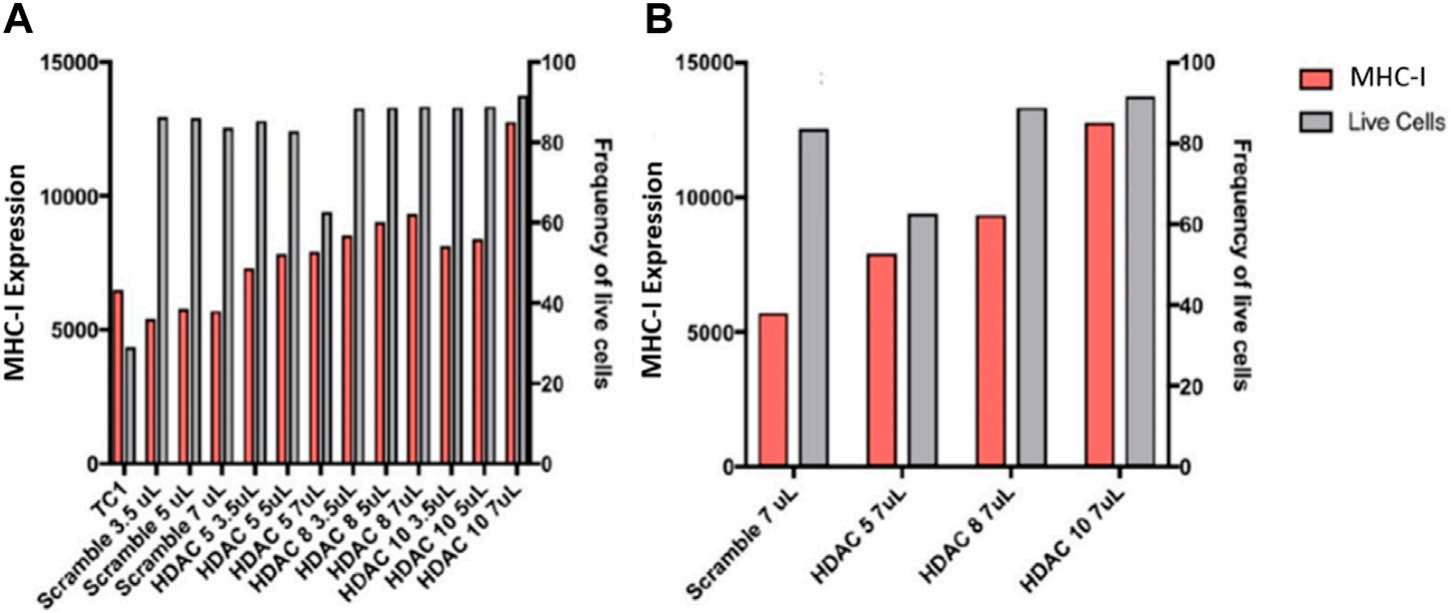
**(A)** Knockdowns of HDACs increase MHC-I expression in TC1 cells. **(B)** MHC-I protein expression is elevated following siRNA treatment of primary TC1 cells against HDACs 5, 8, or 10. The experiments were repeated 3 times (*n* = 3) to accurately calculate the MHC-I expression.

We then proceeded to perform siRNA knockdown of HDACs to test their function in regulating APM expression. Knockdown of HDACs 5, 8, and 10 in TC1 cells, a primary tumour cell line that constitutively express APM components, resulted in an MHC-I surface expression increase, analyzed via flow cytometry analysis (Figure 9). These data support the conclusion that HDACs 5, 8, and 10 individually or collectively regulate APM components in primary tumour cells.

**FIGURE 10:**
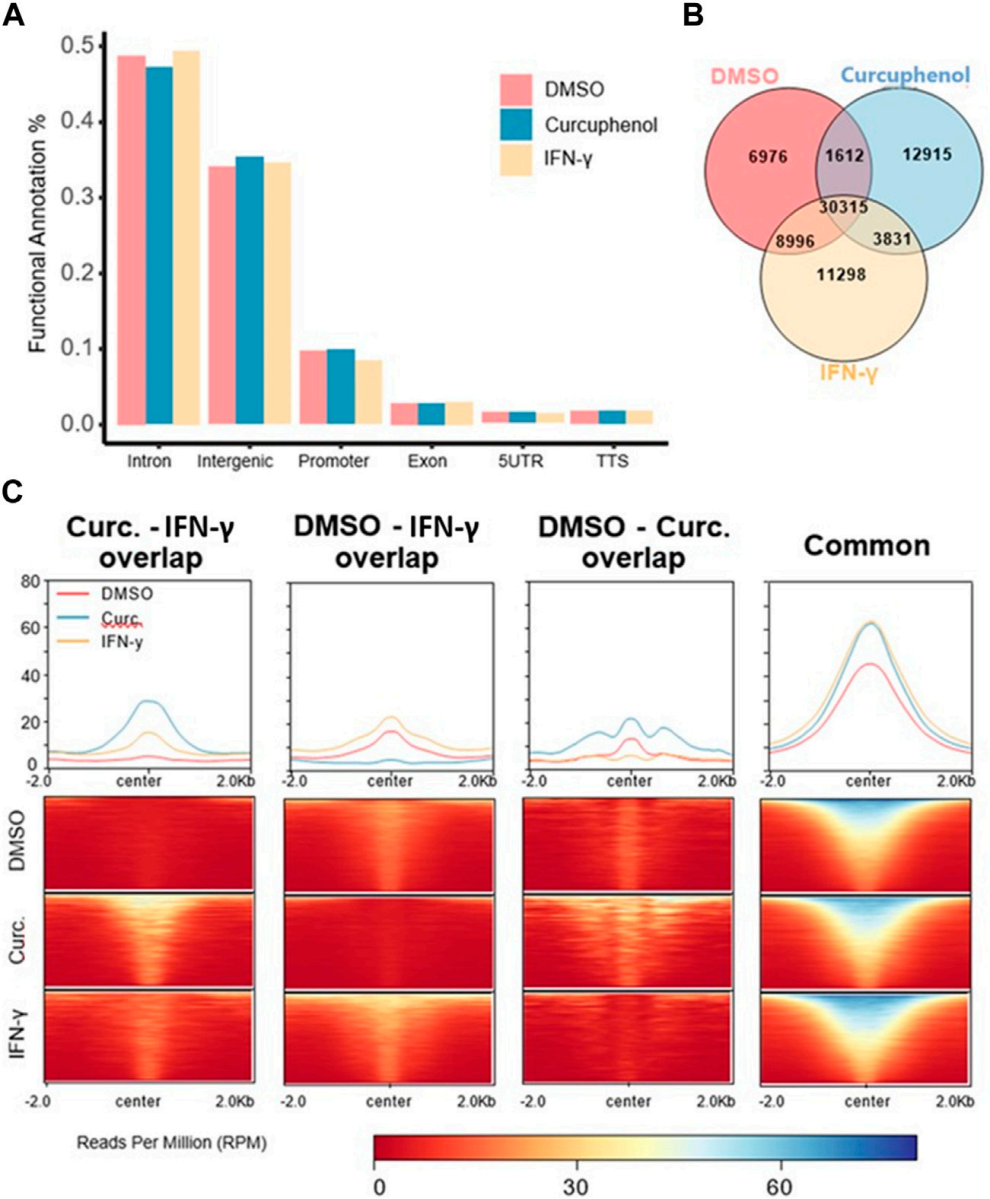
Characterization of H3K27 acetylation induced by curcuphenol treatment of metastatic cells. **(A)** Functional annotation of DMSO, curcuphenol and IFN-γ samples. **(B)** H3K27 acetylation peak locations were compared. **(C)** H3K27 acetylation levels of common regions were higher than all other combinations of interactions. At common regions, curcuphenol (Curc) have increased global acetylation similar to IFN-γ.

To understand how HDAC activators can potentially alter immune evasion *in situ*, we conducted H3K27ac ChIPseq on DMSO-, IFN-γ− and curcuphenol-treated metastatic A9 cells. Functional annotation of H3K27ac regions from all samples demonstrated that the most significant alterations in histone modifications were observed at intronic and intergenic sites suggesting that curcuphenol and IFN-γ alter H3K27ac at enhancer sites (Figure 10A). We also found 40% of acetylation marks were located in H3K27ac regions which are observed commonly in all samples. Interestingly, in this common region set, we observed the effects of curcuphenol were similar to IFN-γ suggesting that both curcuphenol and IFN-γ can modify overall acetylation levels to initiate an immune response. This common region set also contains previously tested MHC genes and their promoters (Figures 10B, C). Despite the similarity in global acetylation levels, we found no relation between IFN-γ and curcuphenol response at the promoter and gene body of these genes. Yet, our expression data clearly demonstrated MHC gene activation by both curcuphenol and IFN-γ, suggesting that curcuphenol and IFN-γ have similar global acetylation responses but have different mechanisms of activation of MHC-I genes.

**FIGURE 11:**
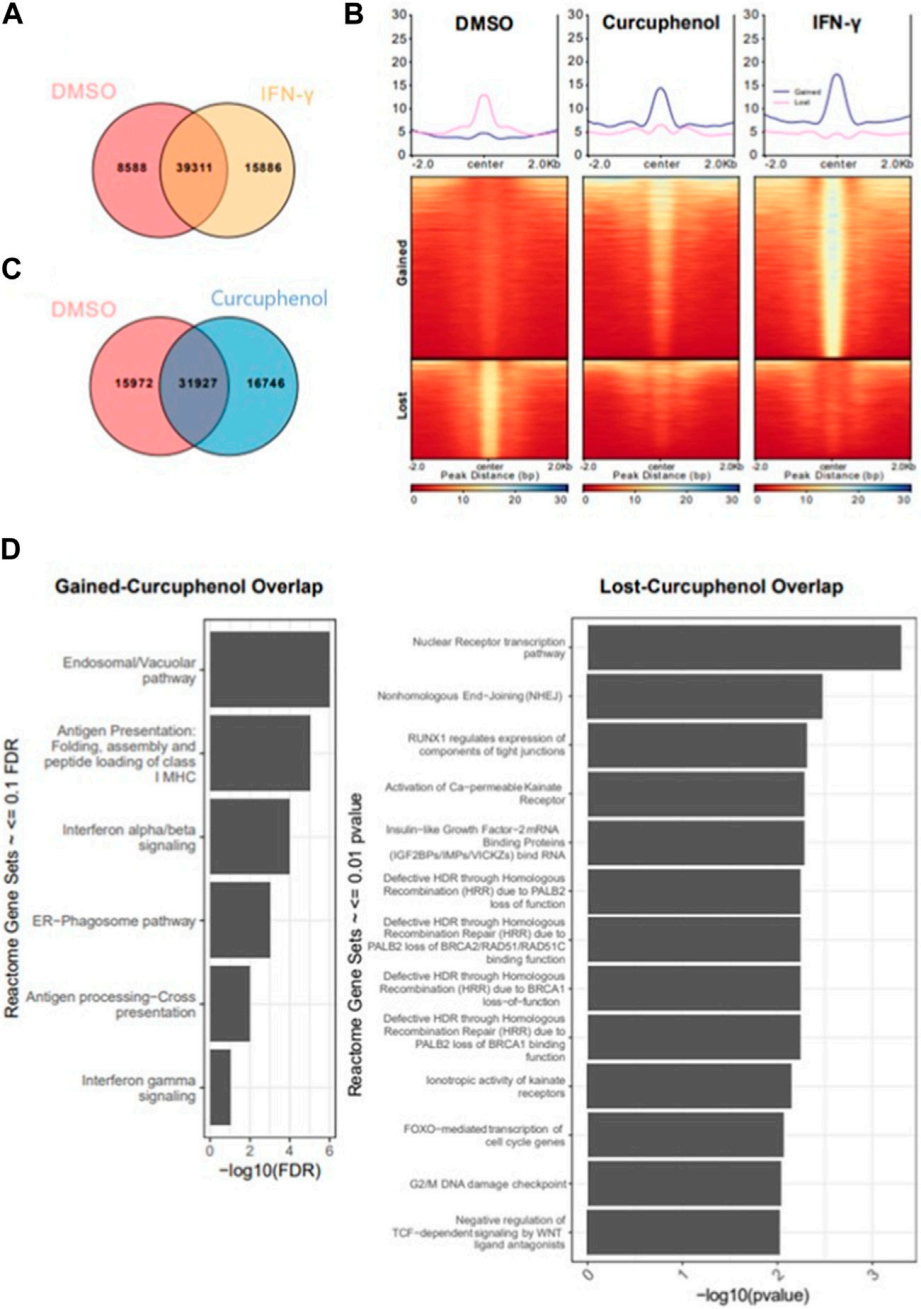
Characterization of cellular pathways effected by curcuphenol treatment of metastatic cells. **(A)** Resulting peaks from ChIPseq analysis were overlapped with DMSO and IFN-γ samples then annotated as DMSO only (Lost, *n* = 8,588), DMSO-IFN-γ common (Common, *n* = 39,311) and IFN-γ only (Gained, *n* = 15,886). **(B)** For gained and lost regions, we compared the H3K27 acetylation levels of each sample. **(C)** Co-occupancy analysis of DMSO and curcuphenol samples. **(D)** Pathway analysis of closest genes with respect to overlap of Gained/Curcuphenol regions was plotted. Gene sets <0.01 false discovery rate (FDR) were filtered out.

Next, we identified acetylation regions either induced or repressed by curcuphenol or IFN-γ treatment with respect to the DMSO sample. We annotated the resulting genomic sets as “gained” (*n* = 15,886), which is specific to IFN-γ and “lost” (*n* = 8,588), which is specific to DMSO. Next, we compared “gained” and “lost” genomic regions with respect to curcuphenol samples to further characterize curcuphenol’s impact on acetylation (Figures 11A–C). 30% (4,588 of 15,886) “gained” regions overlapped with curcuphenol-induced acetylation regions, and 15% (1,612 of 8,588) “lost” regions overlapped with curcuphenol-repressed acetylation. We further conducted gene-set enrichment analysis closest to gene annotation and matching to the human homologs. Consequently, we found that “gained-curcuphenol” regions are associated with known IFN-γ signalling pathways (Nallar and Kalvakolanu, 2014) (Figure 11D-left). Conversely, our data support the conclusion that curcuphenol and its analogues can act as HDAC-activating compounds. Thus, we also examined the loss of H3K27ac marks in curcuphenol-treated metastatic tumour cells compared to the DMSO-treated control. We found that H3K27ac “lost-curcuphenol” regions are enriched with pathways that modulate innate and adaptive immunity as well as tumour cell growth (Figure 11D-right).

## Discussion

Natural product libraries provide an exceptional repository of novel compounds that hold promise as starting points for the creation of innovative therapeutic drugs. These extracts can be derived from everyday food ingredients such as herbs and spices or from remote sources such as deep-sea environments. Although commonly associated with their culinary uses, many spices have been recognized for their therapeutic qualities. Some examples include cumin, saffron, turmeric, green and black tea, and flaxseed, which contain the bioactive compound curcumin (Thompson et al., 1996; Busquets et al., 2001; Abdullaev, 2002; Lai and Roy, 2004). These natural substances offer a valuable resource for drug development and broaden our understanding of cellular processes. Hence, exploring extracts derived from nature continues to be the most significant source for discovering unique therapeutic agents that could potentially inhibit cancer proliferation and impede metastasis. One such compound, curcuphenol, has been extracted from both cumin and turmeric, both widely employed cooking spices that are thought to hold promise in the realm of cancer treatment and prevention by ethnobiologists. By investigating natural sources such as these, we may uncover a wealth of novel compounds that may contribute significantly to the advancement of therapies for cancer and other diseases.

The epigenetic processes includes the covalent modifications to DNA, RNA, and proteins leading to changes in gene expression and is a growing field in cancer research and immunology (Setiadi et al., 2007b; Setiadi et al., 2008a; Concha-Benavente et al., 2016). The first discovered connection made between cancer and epigenetics was the loss of DNA methylation at CpG dinucleotides, and since then, the field has vastly grown to include the modification of other elements such as RNA and proteins as well as other epigenetics alterations currently including acetylation, ubiquitylation, phosphorylation, sumoylation, ribosylation and citrullination (Agrawal and Kishore, 2000; Cock-Rada and Weitzman, 2013; Chen et al., 2015; Li et al., 2021; Song et al., 2021; Xu et al., 2021; Borcoman et al., 2022). Interestingly, inhibitors of HDACs and, to a lesser extent, demethylating agents have shown the ability to induce the expression of APM components (Setiadi et al., 2005a; Setiadi et al., 2008a; Campoli and Ferrone, 2008). HDACs remove acetyl groups from histone tails, imparting a positive charge and increasing the affinity for the negatively charged DNA, causing tightening of local chromatin structure and reduced transcriptional activity. However, the nomenclature of HDAC is misleading as these enzymes are not limited to the deacetylation of histones. HDACs can also remove acetyl groups from other proteins, resulting in a variety of functional effects, including dictating other subsequent post-translational modifications (such as methylation and ubiquitination), increasing protein stability, altering subcellular localization, and altering protein-protein interactions (Feinberg and Tycko, 2004).

Currently, there are eighteen known HDACs, which are divided into four classes, I-IV, based on structure and localization. Class I HDACs are primarily found in the nucleus; they are ubiquitously expressed in various tissues and are thought to play a role in cell proliferation; alternatively, class II HDACs are found in both the nucleus and cytoplasm and are more selectively distributed in tissues (Dawson and Kouzarides, 2012). The class III HDACs known as the sirtuins (Surani et al., 1993; Nguyen and Massagué, 2007b; Escors, 2014) are the most unique as they require nicotinamide adenine dinucleotide for functionality and so far have yet to be affected by any of the current HDAC inhibitors (Dawson and Kouzarides, 2012). The final HDAC group, class IV, contains only a single member, HDAC11, due to its unique structure and prominent localization to the nucleus. Furthermore, physiologically occurring coenzyme A (CoA) derivatives, such as acetyl-CoA, butyryl-CoA, HMG-CoA, and malonyl-CoA, as well as NADPH but not NADP+, NADH, or NAD+, act as naturally available allosteric activators, co-factors or substrates of recombinant HDAC1 and HDAC2 *in vitro*, though the significance of this to epigenetic control in cell and mammalian biology is unknown (Vogelauer et al., 2012). It should be emphasized, that despite their prominance in molecular biology, HDACs may only regulate less than 10% of all genes, and HDAC inhibition can lead to gene repression as well as activation, depending on the nature of the gene (Fass et al., 2003; Glaser et al., 2003; Kato et al., 2004; Nusinzon and Horvath, 2005; Peart et al., 2005).

The antigen-processing genes in many metastatic cancers are under epigenetic control and several pan-HDAC inhibitors have been shown to upregulate APM components, including TSA, Panobinostat, Valporic and Depsipeptide (Setiadi et al., 2005a; Setiadi et al., 2007a; Setiadi et al., 2008a; Ma et al., 2009). However, many of these compounds have not become main stream therapeutics as there are several concerns regarding stability *in vivo* as well as off-target side effects (Rodríguez-Paredes and Esteller, 2011). These difficulties spawned our exploration to determine if curcuphenol analogues have a hitherto undescribed epigenetic modifying activity. To examine this possibility, we established HDAC assays and tested the effect of curcuphenols and its analogues in these HDAC assays. Due to the similar structure of the curcuphenol analogues to a known HDAC inhibitor, TSA, which promotes the expression of MHC-I in the metastatic A9 lung cancer cells (Setiadi et al., 2005a; Setiadi et al., 2007a; Setiadi et al., 2008a), it was predicted that the analogues were acting through a similar mechanism involving by HDAC inhibition. However, upon assessing the activity of curcuphenol analogues using metastatic A9 lung cancer cells in a generalized class I/II HDAC luminescence assay to measure HDAC activity, the opposite effect was discovered, and HDAC activity was observed to be enhanced. This HDAC enhancement is a novel activity that, has never been recorded in the literature for class I/II HDACs.

To determine whether the new curcuphenol analogues P02-113 and P03-97-1 were, in fact, directly interacting with HDAC enzymes to promote activity, individual purified recombinant HDACs were assessed following treatment with the analogues. While most HDAC enzymes did not show a change in activity, two HDACs (5 and 10) exhibited enhanced activity upon treatment with P02-113 and P03-97-1. These are most likely the HDAC candidates showing an increase in activity in the generalized HDAC class I/II assay performed on the A9 cells. This is a unique finding, as HDACs are currently generally viewed as being dysregulated in cancer. The dysregulation of HDACs in tumours leading to an altered gene expression profile, is thought to contribute to the initiation and progression of cancer, as well as to the development of drug resistance in tumours (Tofilon and Camphausen, 2010). However, reductions in the activity of both HDACs 5 and 10 have also been implemented in advanced stages of lung cancer and are correlated with poor outcomes (Osada et al., 2004; Yao and Rahman, 2009).

Interestingly, previous studies that have downregulated HDAC5 using siRNA found that there was a pro-angiogenic effect due to increased endothelial cell migration, sprouting, and tube formation (Urbich et al., 2009). In the case of HDAC10, there has been significantly more research carried out to examine the relationship to its activity in cancer. Decreases in HDAC10 activity have been correlated with more aggressive malignancies in B lymphocyte and have been linked to metastasis in gastric cancer and squamous cell carcinomas (Song et al., 2013; Jin et al., 2014; Powers et al., 2016). Another mechanism has also been suggested for the involvement of HDAC10 in inhibiting metastasis, as it is known to suppress matrix metalloproteases 2 and 9 expression that are critical for cancer cell invasion and metastasis (Song et al., 2013).

In this study, we demonstrate that the knockdown of HDACs 5, 8, and 10 all resulted in an increase in MHC-I cell surface expression in TC1 primary tumour cells. This suggests that the HDACs are involved in regulating MHC-I expression and other APM components through as yet unknown pathways potentially shared by these HDACs, or through different pathways converging on repressing MHC-I expression in primary tumours that constitutively express APM components. The idea that HDACs function through yet undescribed pathways is supported by the abundance of different types of HDACs, their different placements within the cell, and the variation in the genes they target.

In addition, though not the dominating activity, the novel curcuphenol analogues P02-113 and P03-97-1 also inhibited one HDAC enzyme, HDAC8. This is interesting as this is the only HDAC class I HDAC that is known to exist in both the nucleus and cytoplasm and diverged early in evolution from the other HDAC class I enzymes (Chakrabarti et al., 2015). The inhibition of HDAC8 is a unique feature of the compounds, as the majority of HDAC inhibitors that have been developed possess enzymatic activities as pan-HDAC inhibitors. Furthermore, increased HDAC8 activity is known to be associated with several diseases, including neurodegenerative disorders, metabolic deregulation, autoimmune, inflammatory diseases, and cancer (Chakrabarti et al., 2015). Regarding APM expression, it has been demonstrated that HDAC8 acts as a scaffold for cAMP-responsive element binding protein (CREB), a known transcriptional up-regulator of TAP-1 and MHC-I, where upon over-expression of HDAC8, CREB phosphorylation became decreased along with its transcriptional activity (Gao et al., 2009). Furthermore, evidence is mounting that HDACs may co-regulate both immune-escape due to loss of MHC-I expression and the emergence of PD-1/PD-L1 inhibitor refractory tumours (Paul and Sa, 2021; Borcoman et al., 2022).

In search of a molecular mechanism underlying the induction of antigen processing and presentation of metastatic tumour cells, we conducted H3K27ac ChIPseq analysis on curcuphenol-treated A9 metastatic tumour cells in order to understand if curcuphenol acts as an HDAC activator of genes associated with antigen processing and recognition of metastatic cancers. The background for undertaking this analysis was our previous demonstration that HDAC inhibitors induce antigen processing and presentation of metastatic tumours (Setiadi et al., 2005a; Setiadi et al., 2007b; Setiadi et al., 2008a). Here we find that curcuphenol acts as a potent H3K27ac modifier. It has previously been noted that curcumin, in general, possesses an HDAC-inhibiting activity, reviewed in (Soflaei et al., 2018) however, the present study is the first to demonstrate that a specific curcuminoid, curcuphenol and its analogues, can act as HDAC enhancers or inhibitors, depending on the specific HDAC and furthermore, that this effects APM expression. However, it was also remarkable to find that the set of genes that curcuphenol modifies, overlaps considerably with the IFN-γ regulated genes, thereby demonstrating that curcuphenol can act as a potent modifier of immune-related genes. Furthermore, our analysis identified the shared pathways modified by both IFN-γ and curcuphenol, includes genes that specifically regulate MHC-I endogenous antigen processing and presentation but also those involved in antigen cross-presentation, a function previously defined in dendritic cells https://pubmed.ncbi.nlm.nih.gov/22306692/; https://pubmed.ncbi.nlm.nih.gov/14566337/.

As a result of our studies demonstrating that curcuphenol can act as an HDAC activator, we also examined the loss of H3K27ac marks in treated metastatic tumour cells. These results are fascinating because most of the affected pathways are largely transcriptional regulatory pathways associated with immune function and cancer. For example, the FOXO1, a subclass of the fork-head family of transcription factors which are characterized by a distinct fork-head DNA-binding domain, can be triggered in animals unable to respond to immune challenges due to defects in both the Toll and IMD pathways. FOXO1 is also a transcriptional regulator of MHC-II expression and mediates an anti-tumour effect in tumour-associated macrophages (Yang et al., 2018). In addition, the NR4A nuclear receptors, when expressed, act to reduce B lymphocyte responses to antigens when second signals are not present (Tan et al., 2020). Furthermore, PALB2 is a noted tumour suppressor (Pauty et al., 2017). Finally, the Insulin-like growth factors 1 and 2 (IGF-1 and IGF-2) are directly involved in cancer cell expansion, and their bioactivity can be modified by specific IGF-binding proteins (IGFBPs) (Sokol, 2011). Overall, we observe that curcuphenol functions to enhance pathways associated with immune recognition while simultaneously suppressing cancer growth. We deduce that curcuphenol and its analogues serve as epigenetic modulators of immune responses, which clarifies our observation of MHC-I induction by curcuphenol analogues in metastatic cancer cells.

In the present study we observe that curcuphenol and its synthetic analogues possess HDAC-modifying activities, including novel HDAC enhancing activities. In invasive cancers this is linked to reversing the immune-escape phenotype in metastatic tumour cells by epigenetically resurrecting the expression of APM components that trigger adaptive immune responses. This is a provocative finding and serves to highlight the potential medicinal value of components of common spices used as culinary ingredients.

## Materials and methods

### Synthesis of racemic curcuphenol and pharmacophore analogues

Racemic curcuphenol was synthesized to provide sufficient material for biological evaluation, and the curcuphenol structural analogues P02-113 and P03-97-1 were synthesized to increase the bioactivity of the curcuphenol lead structure. The details for synthesis can be found in the *S*upplementary Information.

### TC-1 and A9 cell culture

The primary TC1 tumour cell line, originating from a female C57Bl/6 mouse, was developed by the transformation of murine primary lung cells with the human papillomavirus type16 E6 and E7 oncogenes and activated H-ras (cell division regulating GTP-ase) (Schnare et al., 2001). These cells have high TAP-1 and MHC-I levels (Schnare et al., 2001; Jones and Baylin, 2002; Reiman et al., 2007). The metastatic A9 tumour cells are the metastatic clones derived from the primary TC1 tumour; these cells are capable of metastasis when injected subcutaneously into mice, not just when injected into the blood. The metastatic A9 tumour has downregulated MHC-I expression and APM components (Jones and Baylin, 2002; Reiman et al., 2007). A9 and TC-1 cells were grown in Dulbecco’s Modified Eagle’s Medium (DMEM, Gibco) with 10% Fetal Bovine Serum (FBS, Gibco), 100 U/mL penicillin-streptomycin (Gibco) and incubated at 37°C in a 5% CO_2_ humidified atmosphere.

TSA, curcuphenol, P02-113 and P03-97-1 curcuphenol analogues, and IFN-γ were dissolved in 1% dimethyl sulfoxide (DMSO) (Sigma) in DMEM and 10% FBS as the vehicle. 1 × 10^6^ A9 or TC-1 cells were plated onto a 6-well plate (Corning) in 2 mL of media. A9 cells were treated after 48 h with TSA (100 ng/mL), curcuphenol and curcuphenol analogues (0.02 mg/mL) or IFN-γ (5.832 × 10^−6^ nmol) or 1% DMSO.

### Evaluation of MHC-I surface expression by flow cytometry

Primary TC-1 tumour or metastatic A9 tumour cells were plated in 6-well plates at a concentration of 10,000 cells per well in a 2 mL volume. The following day, cells were treated with TSA (100 ng/mL), curcuphenol and P02-113 and P03-97-1 curcuphenol analogues (0.02 mg/mL), IFN-γ (5.832 × 10^−6^ nmol) or 1% DMSO and incubated for 48 h at 37°C. After incubation, the cells were trypsinized, washed and stained with APC-conjugated anti-mouse MHC- I (specifically anti-H-2K^b^) antibody (clone 25-D1.16; Biolegend) and assessed by flow cytometry analysis. Primary TC-1 tumour cells were used as a positive control for surface MHC-I expression, and vehicle alone (1% DMSO) was used as a negative control.

### Reverse transcription and RT-qPCR

RNA was isolated using the RNeasy plus mini kit. RNA was reverse transcribed into cDNA using the superscript II reverse transcription kit (Invitrogen). Quantitative RT PCR was done using 10 nM of primer and 1 μL of BioRad SYBR Green master mix (Biorad). RT-qPCR was done on 7,500 Fast Real-Time PCR System from Applied Biosystems 40 cycles (95°C denaturing for 15 s, 60°C annealing for 1 min). Primers are as follows Table 1:

**TABLE 1:** Primer sequences used for RT-qPCR.

Primer target	Forward (5’->3′)	Reverse (5’->3′)
HDAC5	5′GCCTCG GAA CCC AAC TTA AA 3′	5′ GGC AGA GAA GGA GAX GTA TAG A 3′
HDAC8	5′CCT GAT TGA CGG GAA GTG TAA 3′	5′GTG CAG GGA CAC AGT CAT AA 3′
HDAC10	5′CCA AAC ATC CCA AGC AGA AATA A 3′	5′CAG CAC CAA CTC AGG ATC AA 3′
TAP1	5′TTA TCA CCC AGC AGC TCA GCC T 3′	5′TCA GTC TGC AGG AGC CGC AA 3′
B-Actin	5′ ATG GAT GAC GAT ATC GCT GC 3′	5′TTC TCC AGG GAG GAA GAT 3′

### Chromatin immunoprecipitation assay (ChIP)

1 × 10^6^ A9 cells were plated onto a 6-well plate in 2 mL of DMEM media. Twenty-four hour after seeding, cells were cultured at the optimum concentrations of curcuphenol (0.00167 μmol), or IFN-γ (5.832 × 10^−6^ nmol) or 1% DMSO vehicle for 48 h. Cell viability was gauged using Trypan Blue dye (Gibco) and cell counting slides (Biorad) with Biorad TC20 automated cell counter. Once the viability was above 90%, the cells were quantified, and 1.3 × 10^5^ live cells were transferred into a new tube. The cells were pelleted at 500 g for 6 min at 4°C. Cells were put into ChIP-seq lysis buffer and mixed up and down three times before being placed immediately in liquid nitrogen for 15–20 s. Cells were then immediately transferred to dry ice and stored at −80°C until they were ready for transport. The sequencing procedure was followed as per the previously published protocol (Lorzadeh et al., 2016).

### Bioinformatic analysis of H3K27ac data

#### Processing of ChIP-Seq Data

Raw sequencing data were aligned to mouse reference genome (mm10) with BWA mem (v0.7.6a) with option (-M). Peak calling was done with MACS2 (v2.1.2) with FDR cutoff 0.01 and option (-f BAMPE) (Feng et al., 2012; Gaspar, 2018). During the peak calling process, input samples were used as background control.

#### Overlap analysis of H3K27ac peaks of DMSO, curcuphenol and IFN- γ samples and Venn diagram

Intervene (v0.6.4) was used to generate a Venn diagram of the comparison with the "--save-overlaps” option to obtain sample-specific or common binding regions (Khan and Mathelier, 2017). Comparison of DMSO and IFN-**γ** generated the region sets lost and gained, which gained represents IFN-γ specific and lost is DMSO specific. Further, gained and lost regions then intersected with curcuphenol regions to create genomic region sets “gained-curcuphenol” and “lost-curcuphenol,” respectively.

#### Heatmap

deepTools (v3.1.3) was used to analyse ChIP-seq data (Ramirez et al., 2014). To generate heatmaps, deepTools’ *computeMatrix* function was used to generate an intermediate matrix file for the heatmap using the following arguments "-a 2000 -b 2000 –reference Point center”, and then used deepTools’ *plotHeatmap* function with default settings.

#### Functional annotation of gained/lost regions

Homer’s (v4.10.3) *annotatePeaks.pl* function was used to annotate all genomic location sets (Heinz et al., 2010). Mouse (*mm10)* genome was used as reference genome while running Homer https://paperpile.com/c/6liArk/SexC.

#### Gene set enrichment analysis

The overlap between “Gained-curcuphenol” and “Lost-curcuphenol” acetylation regions was used to find the closest genes by Homer’s *annotatePeaks.pl* function. Using Metascape tool, corresponding human genes were found for these genes (Zhou et al., 2019). Later, using Reactome Database’s gene set enrichment tools, the enrichment of Reactome pathways was calculated in our gene set. For visualization all the hits less than 0.1 FDR were removed.

#### Data availability

The data in this publication have been deposited in NCBI’s Gene Expression Omnibus (Edgar et al., 2002) and are accessible through GEO Series accession number GSE179844 (https://www.ncbi.nlm.nih.gov/geo/query/acc.cgi?acc = GSE179844).

### HDAC assays

To assess the effect of the compounds on the relative activity of HDAC class I and II enzymes in the A9 cell line, the HDAC-Glo™ I/II Assay and Screening System (Promega) was used. The linear range was established for the A9 cells following the manufacturer’s instructions. 3 × 10^4^ cells per well were plated in clear-bottom 96-well plates (Perkin Elmer), and plates were incubated at 37°C. After 24 h, cells were treated with 25 nM of TSA (positive control), 1% DMSO (negative control), or a range of dilutions of P02-113 or P03-97-1 (0.02–5 μM) and incubated for 30 min. Cell culture media was used as a blank control, and HeLa cells provided in the HDAC assay kit were used as a positive control. HDAC class I/II reagent was then added and incubated for 30 min before luminescence was measured using the Infinite M200 (Tecan) and i-control software (Tecan). To evaluate the effect of the compounds on specific HDACs, their activity was assessed with purified HDAC enzymes from all classes I, II, and IV, as well as a select member of HDAC class III (SIRT1). HDACs 1-9 and SIRT1 were evaluated using HDAC Fluorogenic Assay Kits (BPS Biosciences) following the manufacturer’s recommendations. Compound treatment started at 5 μM and was two-fold diluted to a concentration of 0.02 μM. Alternatively, HDAC 10 and 11 (BPS Biosciences) were optimized to be assayed with the HDAC-Glo™ I/II Assay and Screening System (Promega).

The assays were measured using the Synergy HI hybrid reader (BioTek) and Gen5 software (Bio-Tek). For all assays, vehicle (1% DMSO) was used as a negative control, and TSA (25 nM) was used as a positive control, except for the SIRT1 assay where nicotinamide (5 mM) was used as a positive control. To calculate the fold change in HDAC activity, the values from each treated well were divided by the relative mean of activity of the specific HDAC being measured.

### siRNA knockdowns HDACs in TC1 cells

TC1 cells were plated 24 h before transfection in media (DMEM and 10%FBS) at a concentration of 1 × 10^6^ TC1 cells per well in a 6-well plate. TC1 cells were transfected with 7 μL of siRNAs HDAC5, HDAC8, HDAC10 or Control siRNA-A (Santa Cruz). The siRNA was mixed in 150 μL of Opti-MEM reduced serum medium (Gibco) for 5 min. Concurrently, 12.5 μL of Lipofectamine RNAiMAX (Thermofisher) or Lipofectamine 2000 reagent (Thermofisher) was placed in 150 μL of Opti-MEM for 5 min. The siRNA and lipofectamine in Opti-MEM were then mixed and incubated for 10 min before placing them into each well. Forty-8 h following transfection, TC1 cells were harvested from the plate, and half of the cells were checked for MHC-I expression levels using the anti-mouse H2K^b^ APC antibody (Biolegend) and viability using the 7AAD viability dye by flow cytometry; the other half of the TC1 cells were lysed for quantitative RT-PCR using the RNeasy plus mini kit (Qiagen).

### Statistical analysis

Data were analyzed with R and Excel. A Student’s t-test was used for determining statistical significance between controlled test groups; *p* ≤ 0.05 was considered significant.

## Data Availability

The datasets presented in this study can be found in online repositories. The names of the repository/repositories and accession number(s) can be found below: https://www.ncbi.nlm.nih.gov/, GSE179844.
